# Supplementary material for: Chromothripsis during telomere crisis is independent of NHEJ, and consistent with a replicative origin
Source: Genome Res. 2019 May;29(5):737–49. doi: 10.1101/gr.240705.118 (PMC6499312; doi:10.1101/gr.240705.118)
Supplement: Supplemental Material [file supp_gr.240705.118_Supplemental_file_1.zip › contigs/annotated_contigs/DB81/contig.2.DB81_length_613_mean_cov_9.99673735726.docx]

**DB81_length_613_mean_cov_9.99673735726**

```
AAGCATCATTAGAGTGGCTGAAAACATTTCTTAGGCATAAAACAAACAAAAAACTCGCAAATAACAAGGGCCTGAAAACAGTATAAGCA
 >chr2:189160897-189161162 - E=1e-144
TTGTTTTCTAAGTCTTACTCTGGAACAATGACTTTTAGCCATTCTCTTTAAAAATAAAGGCCTGATTTCAATTTCATATCCCCAGTGTT

TGAAAGTGAAGCGATTTCTCCATTCAAAGTTATACCATTTTTTCCTCTTCCAATCTGAGTATATTTTATGCTCTTATGGGAAAAAGC|A

TTTCCCATAAGAAAAAGCA|TTTCCACTTCCTCACATTCACTCTGTCTAATAATCTCCCTTACTTTAGCACCTAATCACCCATTTTGTA
                    >chr2:189157936-189158264 - E=1e-185
ATACAGACACAAAATCATCAGTATTGTCTCCTGCTAGTCCTAGAAATCAGTCATGACTTGAACATTCTCCGTAACTTTTTATTCTCAAA

TGTTGGTGTAACGTATTTTAAAAGCAGGCACAAAATAAATTTATTAAATCCTTCCTAATTGACTGCATTACACTTTGTCAAACTTGCAG

ATTGAAATAGTGCATGTAAAATGTCTGATTGCAAATGAGTAAGGCGAGCTCAGTTGGGAAACACTACTAGGACCTAGGGAG
```
